# Household smoke exposure risk and acute respiratory infection among children under five years in sub-Saharan Africa: evidence from the demographic and health surveys

**DOI:** 10.1186/s12889-025-24708-7

**Published:** 2025-10-09

**Authors:** Michael Larbi Odame, Rexford Kweku Asiama, Margaret Appiah, Grace Frempong Afrifa-Anane, Frank Kyei-Arthur

**Affiliations:** 1 Department of Sustainable Development and Policy, University of Environment and Sustainable Development, Somanya, Ghana; 2 Department of Sustainable Energy and Resources, University of Environment and Sustainable Development, Somanya, Ghana; 3 DSI/NRF SARCHI Industrial Development, University of Johannesburg, Johannesburg, South Africa; 4 Department of Environment and Public Health, University of Environment and Sustainable Development, Somanya, Ghana

**Keywords:** Acute respiratory infection, Children under five years, Household exposure to smoke, Clean cooking fuel, Unclean cooking fuel, Sub-Saharan africa

## Abstract

**Background:**

Exposure to smoke from unclean fuels increases children’s risk of acute respiratory infection (ARI). Although studies have extensively examined the association between the type of cooking fuel used and ARI in sub-Saharan Africa (SSA), few have accounted for the composite effect of the type of cooking fuel and the place of cooking on ARI. This study examined the effect of household smoke exposure risk on ARI among children under five years in SSA by accounting for the composite effect of the type of cooking fuel and place of cooking. It also examined the covariates of ARI among children under five years in SSA.

**Methods:**

The study used the Demographic and Health Survey (2010–2020) of 33 sub-Saharan African countries. While controlling for household, child and maternal characteristics, logit models were used to examine the effect of household smoke exposure risk on ARI among children under five years, along with their associated odds ratios and marginal effects.

**Results:**

The results showed that the prevalence of ARI among children under five years was 4.4% in SSA. The majority of households (56%) were exposed to a high risk of household smoke exposure. Household smoke exposure risk was a significant predictor of ARI among children under five years. In addition, characteristics of children (sex, age, breastfeeding, and a child living with a mother), mothers (age, education, and marital status), and household (place of residence, wealth index, main floor material and the number of children under 5 years old per household) were significant predictors of ARI among children under five years.

**Conclusions:**

Health practitioners and policymakers should consider these factors when developing interventions to curb ARI among children under five years in SSA.

**Supplementary Information:**

The online version contains supplementary material available at 10.1186/s12889-025-24708-7.

## Background

Pollution associated with the use of unclean fuel remains a significant global problem with severe health consequences. Globally, approximately 2.4 billion people rely on unclean fuels, including wood, dung, crop waste, charcoal, and coal, as their primary energy source for lighting, heating, and cooking [1]. A large proportion of this population is found in low- and middle-income countries (LMICs), primarily in SSA [2, 3].

Exposure to smoke constitutes household air pollution generated by the use of inefficient and unclean fuels in the home. Smoke contains a range of health-damaging pollutants, including small particles that penetrate deep into the lungs and enter the bloodstream [4]. Indoor air pollutants from cooking with unclean fuel cause a wide range of adverse health outcomes, including neonatal mortality, respiratory infections, wheezing, preterm births, low birth weight, and lung cancer [3, 5]. Women and children are disproportionately exposed to indoor air pollutants from cooking with unclean fuel, placing children at a heightened risk of ARI [6, 7]. Global estimates indicate that out of the 6.6 million children under five years who die annually, 95% of the deaths occur in low-income countries, and ARIs account for a third of all these deaths [8].

Children are particularly vulnerable to household air pollution (HAP) due to several factors, including an immature immune system, susceptibility of the developing airways, increased ventilation, and a greater proportion of time spent indoors compared to adults. Air pollution in pregnancy also affects unborn children by impacting lung function, recurrent respiratory tract infections and the development of asthma [9]. Understanding the impact of smoke pollution on children’s health is crucial for the development of policies and interventions to mitigate the effects of smoke pollution on acute respiratory conditions and address the long-term consequences of these exposures.

Despite sustained efforts to lower the burden of ARI in Africa, it remains the leading cause of childhood mortality [10]. In a systematic review and meta-analysis involving 21 studies from Ethiopia, an overall pooled prevalence of 22% of ARI was recorded among children under five years in households where biomass fuel was the main energy source [11]. Additionally, the study revealed a significant association between the use of biomass fuel and ARI in children. Furthermore, other significant factors that significantly predicted ARI among children under five years included being female, the absence of a window in the kitchen, carrying a child on the back or lap during cooking, and a non-separate kitchen from the main house [11].

Similarly, a national study by Shayo and Bintabara [12] in Tanzania found that children exposed to solid biomass fuel were 3 times more likely to develop ARI than those who were not exposed to solid biomass fuel. A study in Nepal assessing the prevalence and factors associated with ARI among children under five years in selected tertiary hospitals of Kathmandu Valley found that the presence of the child in the kitchen while cooking, religious affiliation, and the presence of ARI in the family significantly predicted ARI [13]. However, the study found no significant association between ARI and some child, parental, and household characteristics, such as the child’s age and sex, parental education and occupation, household wealth status, and overcrowding [13].

Although studies have extensively examined the association between the type of cooking fuel use and ARI in SSA [2, 14–18], few studies have accounted for the composite effect of the type of cooking fuel and place of cooking [19–21]. Within the household, cooking takes place in either an enclosed or open area. The cooking location has the potential to reduce the exposure to household air pollution. Well-ventilated cooking locations are expected to retain less smoke, which limits the vulnerability to health outcomes associated with smoke [22–25]. With the availability of Demographic and Health Survey (DHS) data in SSA, we examined the effect of household smoke exposure risk on symptoms of ARI among children under five years in SSA by accounting for the composite effect of the type of cooking fuel and place of cooking. Additionally, we examined the covariates of ARI among children under five years in SSA.

Measuring household smoke exposure risk using a composite variable of the type of cooking fuel and place of cooking provides a more robust measure of household smoke exposure risk compared to using only the type of cooking fuel. Additionally, using a composite variable that combines the type of cooking fuel and place of cooking to measure household smoke exposure risk would enhance researchers’ and policymakers’ understanding of the effect of household smoke exposure on ARI among children under five years old, enabling the design of interventions to address ARI among children under five years in SSA.

## Methods

### Data collection and sources

This study is a secondary analysis of the DHS from 33 sub-Saharan African countries (SSACs) between 2010 and 2020. Table S1 in Additional file 1 lists the countries included in the study. DHS is a cross-sectional, nationally representative survey conducted by the DHS program. A two-stage sample approach was employed to gather data from women aged 15–49 years and men aged 15–59 years. In the first stage, a number of clusters, consisting of selected Enumeration Areas (EAs) in both rural and urban areas, were selected based on a probability proportional to size. In each selected EAs, a listing of households was conducted to get a sampling frame of households. In the second stage, a fixed number of households were selected in each EAs based on equal probability systematic sampling. In all selected households, interviews targeted eligible individuals: women aged 15–49 years and men aged 15–59 years. Among eligible women (15–49 years), those who had children under five years prior to the survey were asked questions about their children. A previous study has provided more information about the sampling procedure and household listing [26].

The DHS covered various demographic and health issues, such as family planning, child health and well-being, and household water and sanitation. The DHS also collected data on household cooking fuel and the location of cooking places. This study focused on all children under five years who lived in households exposed to HAP from using all types of cooking fuels for cooking food at specified cooking places.

The DHS uses different sets of questionnaires to collect a wide range of demographic and health variables for women, children, and men living in sampled households. Using similar data collection tools across different countries makes the DHS one of the best datasets for a study comparing the influence of household populations on child ARI across multiple countries. We obtained permission to download and use this dataset in response to our request to the DHS program on 9 December 2021. Individual countries obtained ethical clearance for their DHS from the Institutional Review Board of ICF (Inner City Fund) International and an ethics committee in their country [27]. Participants from the 33 countries that participated in the DHS also provided written informed consent to participate. In addition, parents or guardians provided written informed consent before participants under 18 years of age were recruited to participate in the DHS.

### Sample size

Moreover, while the dataset contains a substantial number of observations from multiple countries, there is a noticeable imbalance in the distribution of observations due to missing data. The maximum number of observations for a single variable in the dataset is 417,417. Our approach has been to prioritize the selection of relevant independent and outcome variables that yield a sample size with the most possible observations, excluding variables with missing data, those irrelevant to the analysis and variations in the number of observations of selected variables. This approach yields a final sample of 365,830 observations for our regression analysis, which accounts for approximately 87% of the total dataset.

The choice of outcome and independent variables is also supported by a matrix of correlation coefficients, presented in Table S2 of Additional file 1. The results from the table indicate that there is no severe correlation that could cause bias in our regression estimates.

### Measurement of variables

#### Outcome variable

The outcome variable in this study is ARI among children under five years. In the DHS, mothers with children under five years were asked whether their children had been ill with a cough in the two weeks preceding the survey, and those who answered affirmatively were asked whether the cough was accompanied by shortness of breath or rapid breathing problems. Children of mothers who reported that their children had a cough in the last two weeks before the survey, and the cough was accompanied by shortness of breath or rapid breathing problems, were categorized as having symptoms of ARI. Children who had ARI were assigned ‘1’ and those who did not experience a cough or a cough without shortness of breath were assigned ‘0’. Hence, the outcome variable yielded a binary outcome of ‘1’ or ‘0’.

#### Independent variable

Independent variable was identified based on existing literature, which found associations between ARI and some socio-demographic variables. In this study, our analysis was limited to variables consistently measured across all the country surveys.

The main independent variable, household smoke exposure risk (HSER), is a composite variable derived from household cooking fuel types (clean and unclean) and location of cooking places (outdoor and indoor). The World Health Organization (WHO) has categorized cooking fuels as clean for health at the point of use based on (PM) and carbon monoxide (CO) household emissions [28]. Following the WHO criteria, household cooking fuel types were classified as ‘clean’ (electricity, liquefied petroleum gas (LPG), natural gas, and biogas) and ‘unclean’ (kerosene, coal, charcoal, wood, straw, shrubs, grass, and animal dung). The primary focus of this research is to describe the influence of the composite effect of cooking fuel type and cooking location on ARI among children under five years. Therefore, respondents who did not cook food in the household were excluded from the study, as they did not provide information on the type of cooking fuel or place of cooking. This approach is consistent with previous studies, which also excluded individuals who did not cook at home [29, 30].

We defined household smoke exposure risk as the “subjective risk” level based on the expected household smoke exposure associated with the types of cooking fuel and cooking places used [19, 31]. We followed the protocols outlined in a previous study by Ahamad and Tanin [31] to compute the HSER levels. Consequently, the cooking fuel type and the location of the cooking place were combined to generate the exposure variable HSER to obtain four-level mutually exclusive ordered categories from highest to lowest risks of exposure as follows;

i. High HSER (if unclean cooking fuel is used at an indoor cooking place).
ii. Medium HSER (if unclean cooking fuel is used at an outdoor cooking place).
iii. Low HSER (if clean cooking fuel is used at an indoor cooking place).
iv. Very low HSER (if clean cooking fuel is used at an outdoor cooking place).

The measure of household smoke exposure risk accounts for both the types of cooking fuel and the location of the cooking place in household exposure to smoke, which is more robust than using only the type of cooking fuel. Further details for HSER estimation can be found elsewhere [31, 32].

#### Covariates

We also selected our covariates based on evidence from existing literature. Studies have found associations between ARI among children under five years and predisposing factors such as household-level characteristics, maternal characteristics, and child characteristics [8, 11, 33, 34].

Covariates included in the regression models were categorized as follows:

i. Child-level factors: (age of a child, sex of a child, living with mother or not, and initiation of breastfeeding).
ii. Household-level variables (wealth status, place of residence, main floor material and number of children under five years per household).
iii. Mother-level factors (age, education, and marital status).

Covariate measurements are detailed in Table S3 of Additional file 1.

### Descriptive statistics

Summary statistics of the dependent variable and selected covariates can be found in Table S4 of Additional file 1. The variables reflect a range of demographic and socio-economic characteristics, as well as health-related outcomes.

**Fig. 1:**
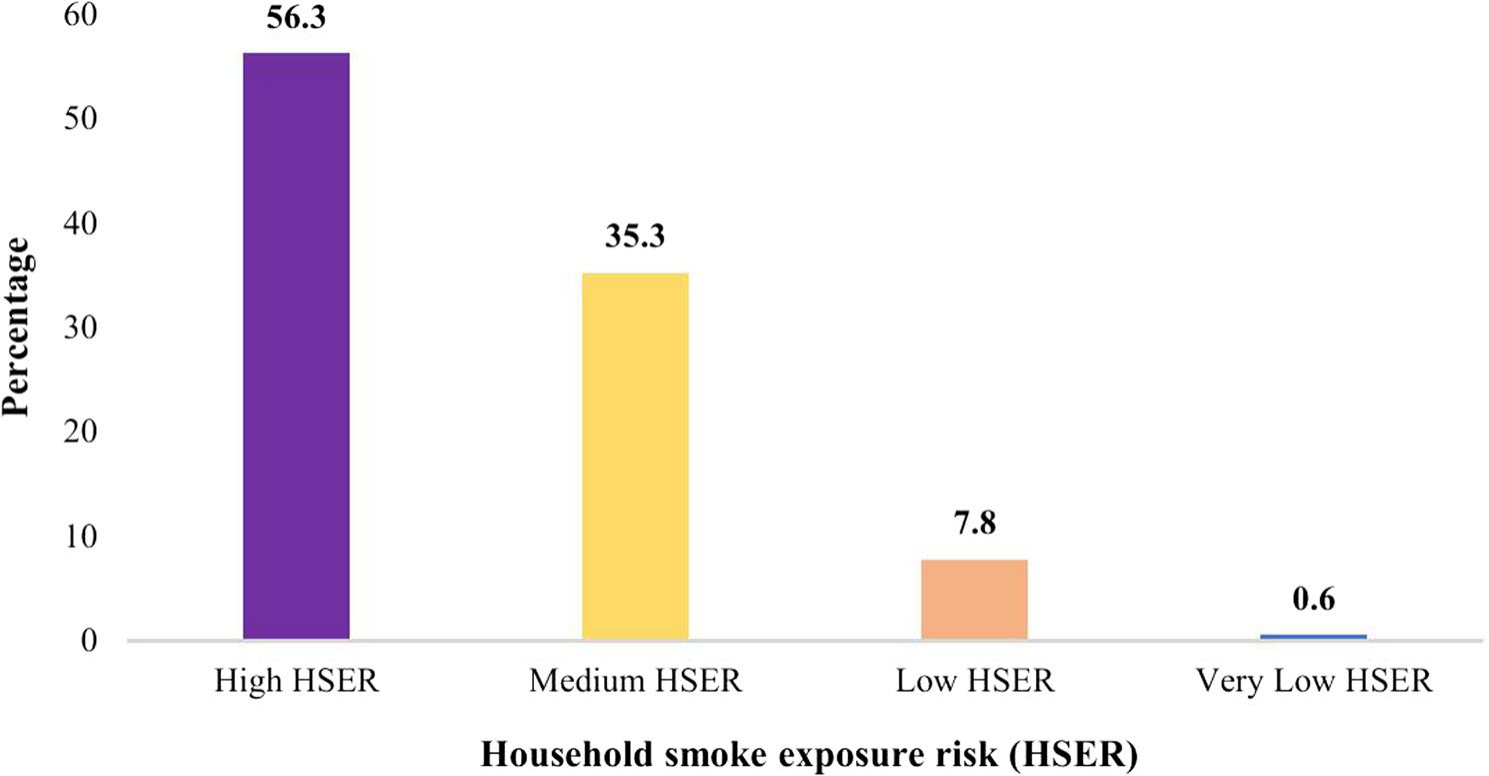
Percentage distribution of household smoke exposure risk

The variable that captures the smoke exposure risk in the household has a mean value of 1.526, indicating that several households in the sample experience a medium to high smoke exposure risk. Further frequency analysis showed that more than 50% of households reported incidences of high smoke exposure risk (See Fig. 1). It is on this premise that the study bases its empirical investigation.

Furthermore, variables which denote whether the child lives with their mother and whether the mother has received education, respectively, exhibit relatively low mean values (0.137 and 0.395). These results suggest that a smaller portion of children in the sample live with their mothers, while a reasonable proportion of mothers have attained some level of education (high school and above).

Additionally, the variable representing the initiation of breastfeeding has a mean of 1.68, indicating that most mothers in the sample initiate breastfeeding within the first day. The mother’s age follows a similar pattern, with a mean of 1.947, indicating that mothers in the sample tend to be in the older age groups.

The socio-economic variable, wealth index, exhibits a mean of 1.85, indicating a concentration toward the middle wealth brackets. This is further supported by the main floor variable, which captures the material of the main floor in the household. With a mean of 1.459 and minimal variation, this variable suggests that most households have similar flooring materials.

In addition, the variable representing the number of children under five years in respondents’ households showed an average of 0.649, indicating that about 65% of the households in the data reported having more than one child under five years. The remaining 35% of households in the data reported having one child under five years.

The variable, representing the urban or rural setting, has a mean of 1.686, indicating that the sample predominantly consists of individuals residing in urban areas.

Overall, these descriptive statistics highlight essential patterns in the data, including a high prevalence of smoke exposure risk, moderate educational attainment, and wealth distribution skewed toward higher categories. These factors will be crucial in the subsequent regression analysis as we explore the relationships between these variables and the outcomes of interest.

### Model specification and data analysis

ARI among children under five years was coded as a binary outcome: ‘1’ for children who had ARI and ‘0’ for those who did not. Therefore, we employed a logit regression model to generate odds ratios and marginal effects, examining the influence of selected covariates on ARI among children under five years. Unlike previous studies [19] that have used only odds ratios, we choose to include marginal effects to illustrate the change in probability resulting from a unit change in the covariates [35]. Marginal effects are estimates of probability and are therefore expected to range between 0 and 1, unlike odds ratios, which have no significant units and only indicate the direction of change in probability.

The associated marginal effects of the logit regression model are obtained by calculating the ratio of the probabilities of a child contracting ARI versus the probability that the child is free of ARI. This can be expressed in Eq. (1) as:

1 $$\:\frac{{P}_{i}}{1-\:{P}_{i}}=\:\frac{1+\:{e}^{{(X}_{i}^{{\prime\:}}\beta\:)}}{1+{e}^{{-(X}_{i}^{{\prime\:}}\beta\:)}\:}=\:{e}^{{(X}_{i}^{{\prime\:}}\beta\:)}\:\:\:$$

Taking logarithms of both sides yields the following equation:

2 $$\:\text{log}\left[\frac{{P}_{i}}{1-\:{P}_{i}}\right]=\:\frac{1+\:{e}^{{(X}_{i}^{{\prime\:}}\beta\:)}}{1+{e}^{{-(X}_{i}^{{\prime\:}}\beta\:)}\:}=\:{Y}_{i}=\text{log}\left[{e}^{{(X}_{i}^{{\prime\:}}\beta\:)}\:\right]$$

Equation (2) is now simplified to obtain the probability model to be estimated based on a vector of coefficients and control variables of the respondents:

3 $$\:{Y}_{i}=f\left({X}_{i}^{{\prime\:}}\beta\:\right)$$

With the binary dependent variable being the probability of a child getting ARI, Eq. (3) is therefore rewritten as:

4 $$\:{P}_{r}\left(Infections\left[C\right]1|X\right)=\:{\beta\:}_{0}+\:{\gamma\:}_{i}{HSER}_{i}+\:{\beta\:}_{i}{Z}_{i}+{\mu\:}_{i}+\:{\epsilon\:}_{i}$$

Where $\:{P}_{r}\left(Infections\left[C\right]1|X\right)$ is the probability of a respondent’s child getting ARI from smoke emissions, and *HSER*_*i*_ represents the levels of HSER (low, very low, medium, high) captured in the dataset. $\:{\mu\:}_{i}$ captures country invariant fixed factors such as the regions identified across countries during the survey, while $\:{\epsilon\:}_{i}$ represents the random disturbance term.

Furthermore, the vector $\:{Z}_{i}$ is used to capture other covariates that influence the likelihood of ARI among children. These covariates are human and physical characteristics of respondents and their households who participated in the survey. Specifically, we consider characteristics of children, mothers and their households as additional covariates and control for their influence in the model estimates.

Regarding child characteristics, we consider factors such as the child’s age, when breastfeeding was initiated, and whether the child lives with the mother. Regarding the characteristics of the mother, we consider the age of the mother, the mother’s education, as well as the marital status of the mother.

Finally, the characteristics of the household considered include the household’s wealth index (rich, middle-income, or low-income), the location of residence (urban or rural), the material of the main floor of the housing structure, as well as the number of children under five years per household.

The paper used descriptive techniques, such as simple frequencies and percentages, to describe the background characteristics of the sample and estimated a standard logistic regression model specified in Eq. (4). In addition to the covariates, the regressions also account for country fixed effects by including regional classifications across countries. These fixed effects address unobserved heterogeneity and confounding effects. All variables are considered statistically significant at a 95% confidence interval (ci) (*p* < 0.05).

## Results

### Socio-demographic characteristics of the study population

The socio-demographic characteristics of the study population are illustrated in Table 1. According to Table 1, most of the children (69%) lived in rural areas and were aged one year or older (79%). In terms of household characteristics, 48% of households were from poor households, while 46% of households had their main floor material classified as unimproved. Also, most households (65%) had more than one child under five years.

Regarding the mothers, approximately 61% had attained an education level below high school, and about 86% were married (Table 1). Also, 76% of mothers were under 35 years. Regarding the children, 50.6% of them were males, and most (86%) lived with their mothers at the time of the survey.

**Table 1:** Socio-demographic characteristics of the study population

	Frequency	Percentage
Sex of child*		
Male	204,314	50.61
Female	199,420	49.39
Child’s age**		
0–12 months	80,891	21.44
1 year and older	296,413	78.56
Child lives with mother		
Yes	360,182	86.29
No	57,235	13.71
Mother’s education		
Below high school	252,523	60.50
High school and above	164,872	39.50
Initiation of breastfeeding		
Immediately	207,920	49.81
Within first day	135,044	32.35
After first day	74,453	17.84
Mother’s age		
Below 35	318,162	76.22
35–49	99,255	23.78
Marital status		
Never in union	30,372	7.28
Currently in a union/living with a man	358,393	85.86
Formerly in a union/living with a man	28,652	6.86
Wealth index		
Poor	199,088	47.70
Middle	81,663	19.56
Rich	136,666	32.74
Place of residence		
Urban	131,135	31.42
Rural	286,282	68.58
Main floor material		
Unimproved	225,872	54.12
Improved	191,480	45.88
Number of children under 5 years per household		
One child under 5 years	146,351	35.06
More than one child under 5 years	271,066	64.94
Total	417,417	100.00

^*^n = 403,734

^**^n = 377,304

**Fig. 2:**
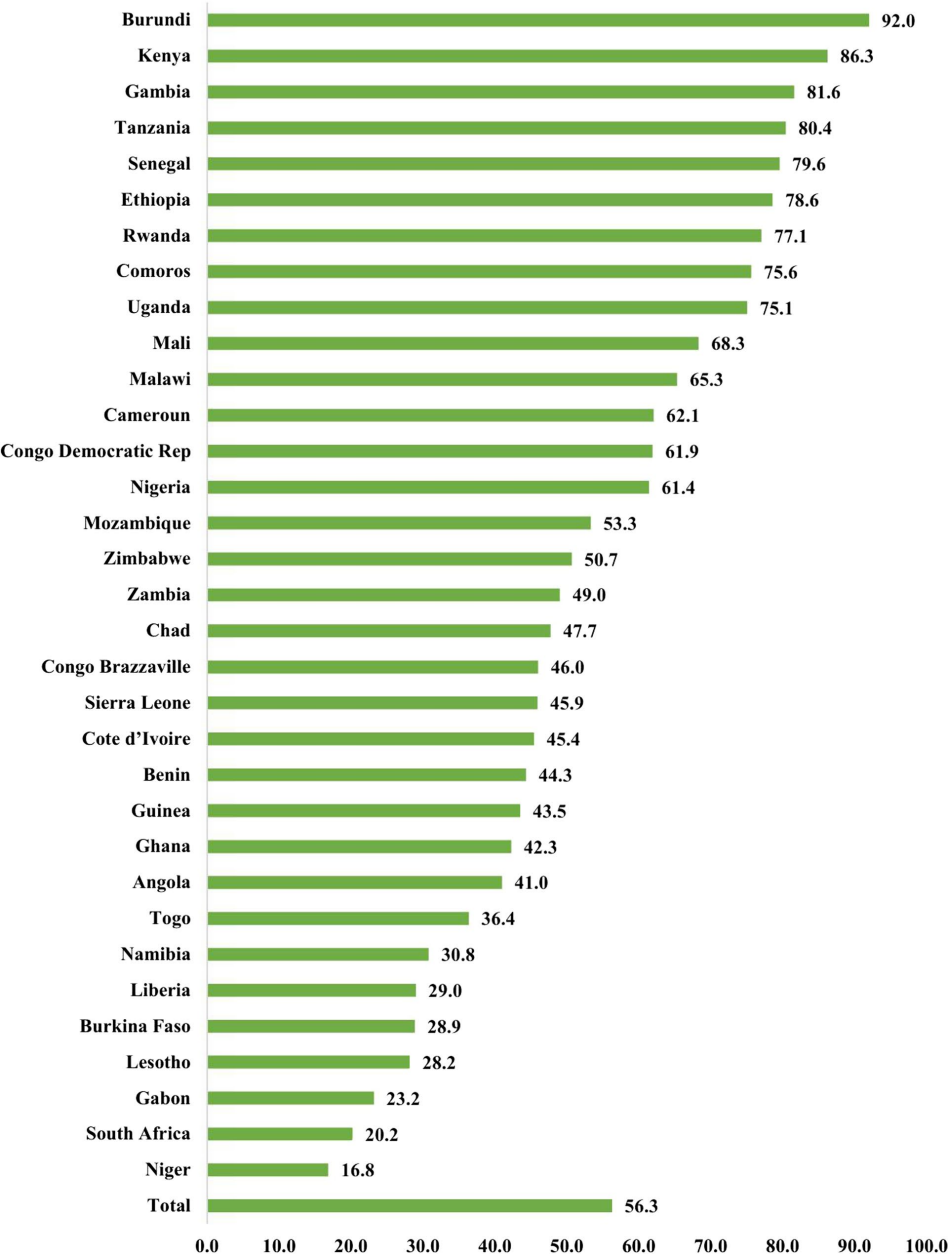
Percentage distribution of high household smoke exposure risk

Regarding HSER, more than half of the households (56.3%) were exposed to high HSER, whilst only 0.6% of the children living in households were exposed to very low HSER (Fig. 1). In terms of high HSER, the prevalence ranged between 16.8% and 92.0%. On the one hand, the top-five SSACs with high HSER were Burundi (92.0%), Kenya (86.3%), Gambia (81.6%), Tanzania (80.4%) and Senegal (79.6%). On the other hand, the bottom-five SSACs with high HSER were Niger (16.8%), South Africa (20.2%), Gabon (23.2%), Lesotho (28.2%), and Burkina Faso (28.9%) (Fig. 2).

**Fig. 3:**
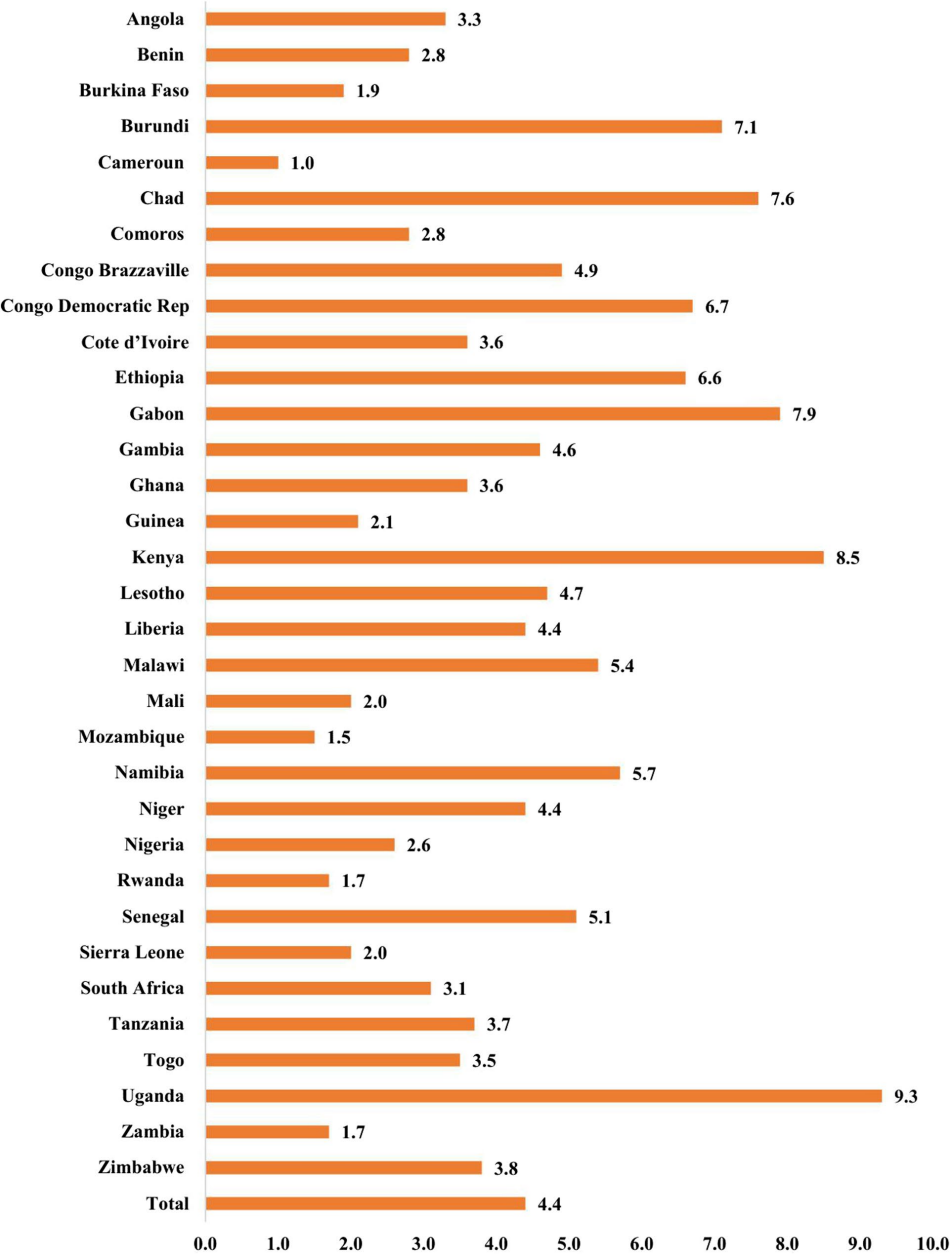
Prevalence of ARI among children under five years in SSACs included in the study

Figure 3 illustrates the prevalence of ARI among children under five years in the 33SSACs included in the study. The total prevalence of ARI among children under five years is 4.4%. The prevalence ranges from 1.0% in Cameroon to 9.3% in Uganda. Fourteen of the thirty-three countries had an ARI prevalence of more than 4.4%.

### Understanding the drivers of ARI among children under five years – the effect of SER, child, mother and household characteristics

Due to the small sample size of the very low HSER category, it was merged with the low HSER category and collectively labeled as ‘low HSER’. The results from the estimation of the empirical model are presented using odds ratios (Tables 2 and 3) and marginal effects (See Tables S5 and S6 in Additional file 2). The results in both tables confirm that HSER was significantly associated with ARI among children under five years. According to Table 2, HSER accounts for 0.4% of the variation in child ARI infections. When covariates are included alongside HSER, the explained variation increases to 1.65% (Table 3).

**Table 2:** Effect of HSER on child ARI infections (odds ratios)

	(1)	(2)	(3)	(4)
Variables	odds ratios	aster	ci	se
Smoke Exposure Risk = = Low (RC)				
Smoke Exposure Risk = = Medium	1.022		0.959–1.090	0.033
Smoke Exposure Risk = = High	1.230	^***^	1.157–1.307	0.038
Constant	0.036	^***^	0.024–0.053	0.007
Observations	365,901			
Dependent Variable	Child ARI Infections			
Wald chi2	509^***^			
Pseudo R2	0.004			
Regional Fixed Effects	Yes			

*RC* Reference category, *ci* 95% confidence intervale, *se* Standard errors

^*^p < 0.1

^**^p < 0.05

^***^p < 0.01

**Table 3:** Effect of HSER, child, mother and household characteristics on child ARI infections (odds ratios)

	(1)	(2)	(3)	(4)
Variables	odds ratio	aster	ci	se
Smoke Exposure Risk = = Low (RC)				
Smoke Exposure Risk = = Medium	0.943	*	0.880–1.010	0.033
Smoke Exposure Risk = = High	1.083	**	1.013–1.157	0.037
Sex of Child = = Male (RC)				
Sex of Child = = Female	1.065	***	1.032–1.100	0.017
Child’s age = = 0 (0–12 months) (RC)				
Child’s age = = 1 (1 year or more)	0.875	***	0.843–0.909	0.017
Lives with mother = = Yes (1)	0.327	***	0.289–0.371	0.021
Lives with mother = = No (0) (RC)				
Education of mother = = 0 (Below high school) (RC)				
Education of mother = = 1 (High school and above)	0.743	***	0.717–0.770	0.013
Initiation of breast = = 1 (Immediately) (RC)				
Initiation of breast = = 2 (Within first day)	1.295	***	1.249–1.343	0.024
Initiation of breast = = 3 (After first day)	1.620	***	1.552–1.690	0.035
Marital status = = 0 (Never in union) (RC)				
Marital status = = 1 (Union/living with a man)	1.112	***	1.038–1.192	0.039
Marital status = = 2 (Formerly in union)	1.221	***	1.116–1.335	0.056
Age of mother = = 1 (Below 35 years) (RC)				
Age of mother = = 3 (35–49 years)	0.951	***	0.917–0.986	0.018
Wealth index = = 1 (Poor) (RC)				
Wealth index = = 2 (Middle)	0.926	***	0.886–0.968	0.021
Wealth index = = 3 (Rich)	0.884	***	0.843–0.928	0.022
Main floor material = = 1 (Unimproved) (RC)				
Main floor material = = 2 (Improved)	0.787	***	0.757–0.819	0.016
No. of children under 5 in household = = 1 (One child) (RC)				
No. of children under 5 in household = = 2 (More than one)	0.898	***	0.867–0.929	0.016
Place of residence = = 1 (Urban) (RC)				
Place of residence = = 2 (Rural)	1.072	***	1.025–1.120	0.024
Constant	0.058	***	0.039–0.089	0.012
Observations	365,830			
Dependent Variable	Child ARI Infections			
Wald chi2	2025***			
Pseudo R2	0.0165			
Regional Fixed Effects	Yes			

*RC* Reference category, *ci* 95% confidence interval, *se* Standard errors

^*^p < 0.1

^**^p < 0.05

^***^p < 0.01

From Table 3, relative to children exposed to low HSER, children exposed to High HSER were more likely to contract ARI infections (OR = 1.083, 95% ci = 1.013, 1.157). However, compared to children exposed to low HSER, children exposed to medium HSER were less likely to contract ARI infections (OR = 0.943, 95% ci = 0.880, 1.010). This result suggests that with medium exposure, the risk of ARI is lower because households in this category use polluting fuels outdoors.

Concerning the characteristics of the child, the results obtained showed that female children are more likely to contract ARI infections (OR = 1.065, 95% ci = 1.032, 1.100) relative to male children, as female children seem to spend more time with their mothers or caregivers fulfilling culinary tasks. On the other hand, the results show that children living with their mothers were less likely to contract ARI infections (OR = 0.327, 95% ci = 0.289, 0.371) compared to those who do not live with their mothers. Similarly, children aged 1 year and older were less likely to contract ARI infections (OR = 0.875, 95% ci = 0.843, 0.909) compared to children aged younger than one year.

With maternal characteristics, the results obtained show that children whose mothers started breastfeeding them within the first day (OR = 1.295, 95% ci = 1.249, 1.343) and after the first day (OR = 1.620, 95% ci = 1.552, 1.690) were more likely to contract ARI infections compared to mothers who started breastfeeding their children immediately. Regarding maternal education, children whose mothers had attained high school or higher education (OR = 0.743, 95% ci = 0.717, 0.770) were less likely to contract ARI infections compared to those whose mothers had attained below high school education.

Furthermore, children whose mothers were currently in a union (OR = 1.112, 95% ci = 1.038, 1.192) and those who were formerly in a union (OR = 1.221, 95% ci = 1.116, 1.335) were more likely to contract ARI infection relative to children whose mothers had never been in a union. Conversely, children of mothers aged 35 and 49 years were less likely to contract ARI infections (OR = 0.951, 95% ci = 0.917, 0.986) compared to children whose mothers were aged below 35 years.

We further find that some household characteristics were significantly associated with the probability of children contracting ARI infections, as displayed in Table 2. First, the results show that children residing in rural areas were more likely to contract ARI infections (OR = 1.072, 95% ci = 1.025, 1.120) compared to children living in urban areas.

In addition, the results show that children from households with more than one child under five years were less likely to contract ARI infections (OR = 0.898, 95% ci = 0.867, 0.929) compared to children from households with a single child. Likewise, children from households with improved main floor material were less likely to contract ARI infections (OR = 0.787, 95% ci = 0.757, 0.819) compared to children from households with unimproved main floor material.

Furthermore, children from middle-income households were less likely to contract ARI infections (OR = 0.926, 95% ci = 0.886, 0.968) compared to children from poor-income households. Correspondingly, children from rich-income households were less likely to contract ARI infections (OR = 0.884, 95% ci = 0.843, 0.928) compared to children from poor-income households.

## Discussion

This study examined the effect of HSER on ARI among children under five years in SSA, controlling for known characteristics of such children, their mothers, households, and country-fixed factors. This study departs from previous multi-country studies that examined the independent effect of household cooking fuel and place of cooking on ARI among children under five years [2, 14–18]. Our findings suggest that reducing household smoke exposure lowers the risk of ARI in children under five years. This finding aligns with previous studies, which found that children under five years who were exposed to high HSER were more likely to develop ARI than those with low exposure [20, 21]. This is because children in households that use unclean cooking fuel in an indoor cooking place (high HSER) are exposed to higher harmful pollutants than those exposed to medium and low HSER. Smoke from unclean cooking fuel contains harmful pollutants, such as PM and NO_x_, which increase the risk of ARI [36, 37]. In this context, households at high risk of smoke exposure may require assistance in transitioning from unclean to clean cooking fuels. Policymakers can facilitate this by making clean cooking fuels more affordable, as affordability has been identified as a barrier to the use of clean cooking fuels, such as LPG [38]. Such an intervention is necessary if SSACs aim to achieve SDG 7.1. Additionally, policymakers could scale up the recent advocacy to promote the adoption of improved and less expensive biomass cooking stoves (ICS), as they tend to have lower particulate smoke emissions. These stoves are more energy-efficient because they burn biomass fuels more thoroughly, resulting in lower emissions [39, 40].

Additionally, this study found that children exposed to medium HSER were less likely to develop ARI compared to those exposed to low HSER. The finding suggests that cooking outdoors with unclean cooking fuel yields a better health outcome than cooking indoors [22, 24, 41, 42], indicating that the health effects of unclean cooking fuel may be moderated by the location where food is cooked. Yet, most studies on the effect of household pollution on children’s health in SSA have neglected the critical role of the setting in which cooking occurs. This study, therefore, contributes to the emerging body of research advocating that discussions on household air pollution resulting from exposure to cooking fuel should not be isolated to the place where food is cooked in SSA [24]. Switching to outdoor cooking from indoor cooking has been observed to have beneficial health implications, particularly in terms of ventilation and reduced smoke exposure. According to Lenz et al. [25], kitchen ventilation interventions could reduce particulate smoke concentration nearly as much as many real-world clean stove interventions. We, therefore, propose to policymakers in the sub-region to promote outdoor cooking by reviewing existing housing policies to encourage investments in housing designs that include well-ventilated cooking areas.

The study’s findings revealed that the inclusion of covariates alongside HSER increased the explained variation in child ARI infections from 0.4 to 1.65%. This suggests that incorporating child, mother and household characteristics improved the model’s explanatory power. Future studies should explore additional covariates, such as environmental, geographic, and health system factors, to further enhance the model’s ability to account for variation in child ARI infections.

This study’s findings also show that female children were more likely to develop ARI than male children when exposed to the risk of household smoke exposure. This finding is consistent with systematic reviews and meta-analyses conducted in SSA, which found a higher likelihood of ARI among female children than male children [11, 41]. Culturally, females tend to spend more time with their mothers when they are cooking compared to male children. This increases the exposure of female children to harmful pollutants associated with cooking smoke, thereby increasing their risk of ARI [11]. This finding highlights the urgent need for public health education, encouraging parents to keep their children under five years, especially females, away from cooking areas to reduce their risk of developing ARI.

With regard to the age of children, children older than a year had a decreased likelihood of developing ARI relative to children aged less than 12 months, which aligns with previous studies in Pakistan and Bangladesh [33, 36]. Khan & Lohano’s [36] study in Pakistan found that children aged more than 24 months were less likely to develop ARI than younger children. Also, Imran et al.‘s [33] study in Bangladesh found that Younger children were more likely to develop ARI than older children aged 48–59 months. A probable explanation is that as children age, their immune responses improve [42], which reduces their risk of experiencing adverse health outcomes, such as developing ARI. This finding implies that younger children are a vulnerable population when it comes to ARI, so policymakers should target mothers with younger children when implementing measures to reduce ARI among children under five years.

Regarding breastfeeding, children who were immediately put to the breast were less likely to develop ARI relative to those who were breastfed within the first day of birth. This finding highlights the protective role of early breastfeeding initiation within the first hour of birth against illness such as pneumonia, ARI, and diarrhea, as well as infant mortality [43, 44]. Policymakers should consider promoting early breastfeeding initiation as part of measures to address ARI among children under five years.

This study found that children of mothers who have never married had a lower likelihood of ARI than children of mothers who are currently in a union or formerly in a union. Never-married women have the autonomy to make healthcare decisions on their own [45], including seeking healthcare services for their children. This autonomy can positively impact their children’s health outcomes, potentially reducing their children’s risk of ARI.

Children who were living with their mothers were less likely to develop ARI relative to those who were not living with their mothers. This aligns with our expectations, as mothers are often primary caregivers who provide proper care and possess adequate knowledge about their children’s welfare, compared to other caregivers.

Consistent with other studies [8, 20, 36, 46], the education of mothers was identified as a significant predictor of ARI. High school or higher education has been shown to positively influence the initiation of breastfeeding [47, 48]. Furthermore, breastfeeding, especially exclusive breastfeeding, is associated with reduced risks of ARI and ARI-related mortality [49–51]. This may help explain why children of mothers with at least high school are less likely to contract ARI. Policymakers should prioritize outreach to women with less than a high school education, as their children are more vulnerable to contracting ARI.

Furthermore, the results suggest that children residing in rural areas were more likely to contract ARI. This finding supports Kilabuko and Nakai’s [52] study in Tanzania, which found that children who resided in rural residences were at a higher risk of contracting ARI due to inadequate access to medical health care and poor socio-economic status. These conditions in rural settings make households highly dependent on unclean sources of fuel [14]. To reduce the risk of ARI among children residing in rural areas, policymakers should enhance access to quality healthcare services in these areas and improve housing conditions.

Also, similar to other studies [53, 54], children from middle-income and rich-income households had a decreased likelihood of ARI relative to children from poor households. The risk of ARI in children may be reduced by improved living conditions and access to healthcare services, which are associated with higher household wealth indices (middle and rich) [53]. Policymakers implementing pro-poor interventions to improve access to quality healthcare services can help reduce the risk of ARIs among children from low-income households.

In addition, households with more than one child under five years were less likely to have children develop ARI relative to those with one child under five years. A plausible explanation is that households with more than one child under five years may have more experience with childcare, which could reduce the risk of children in those households developing ARI.

Regarding main floor material, children in households with improved main floors were less likely to develop ARI than those from households with unimproved main floors. This finding supports previous studies, which found that improved floor material reduces childhood morbidity [43, 55]. A household’s unimproved main floor could easily become contaminated, increasing children’s risk of developing ARI [56]. Given this finding, policymakers should consider subsidizing improved floor materials, such as cement and tiles, to facilitate their uptake, especially among low-income households. Moreover, public health campaigns on the importance of household hygiene, especially regular floor cleaning, should be strengthened to mitigate the risk of ARI among children.

### Strengths and limitations

This study accounts for the types of cooking fuel and the location of the cooking place in relation to household exposure to smoke. Our measure of household exposure to smoke is more robust than using only the type of cooking fuel, and consequently, provides a better understanding of the levels to which household members are exposed to the risk of smoke pollution from cooking food. In addition, the study utilized large, nationally representative data from 33 SSACs, where the prevalence of ARI and use of unclean cooking fuels are high. Hence, the findings of our study can be generalised. However, our study has some limitations. First, the DHS datasets are cross-sectional. Therefore, causality cannot be established between the dependent and independent variables. Thus, future studies could consider the use of longitudinal data. Second, ARI cases were self-reported, so there is a possibility that respondents may provide inaccurate responses due to recall bias or social desirability. Third, the DHS of the 33 SSACs were conducted between 2010 and 2020. Hence, the study’s findings should be interpreted with caution due to the differences in the data collection periods.

Fourth, we could not control for potentially confounding variables such as ambient air pollutants, seasonal variations in environmental temperatures and humidity, second-hand smoking, kitchen ventilation, and frequency of cooking. These variables were not adequately captured during the DHS or were found missing in the data of many of the countries used in the study. Consequently, some confounding variables that may influence ARI among children remain unaccounted for. Also, this study did not include information on the level or duration of household smoke exposure since it was not captured in the DHS. Additionally, we acknowledge the measure of HAP as a limitation in this study. HSER is a subjective measure of household members’ exposure to air pollution, based on self-reported choices of cooking fuel type and cooking place. An objective measure of actual exposure levels to emissions from cooking smoke is recommended in future studies. Moreover, respondents may misinterpret questions during the data collection, which can introduce measurement errors. To minimize these errors, the DHS program employs standardized questions and procedures. Additionally, enumerators recruited to collect the DHS data undergo rigorous training.

## Conclusions

Overall, this study demonstrates that exposure to high levels of cooking smoke significantly increases the likelihood of ARI among children under five years in SSA. The use of unclean cooking fuels in enclosed environments poses a serious health risk, compounding the vulnerability of young children. Beyond environmental exposure, the study highlights that a range of child, maternal, and household characteristics—including the child’s age, sex, initiation of breastfeeding, and whether the child lives with the mother; the mother’s age, education, and marital status; and household indicators such as wealth index, flooring material, and number of young children—are significantly associated with the risk of ARI.

These findings underscore the need for multifaceted interventions that go beyond improving household energy use. Health practitioners and policymakers must consider these factors when designing strategies to reduce ARI incidence. Also, the high prevalence of ARI among some SSACs points to the urgent need to strengthen domestic pharmaceutical manufacturing in some SSACs. Building local capacity to produce essential paediatric medicines—such as antibiotics and respiratory treatments—would improve access to timely care, reduce dependence on external supply chains, and ensure formulations are suited to local needs. This approach will be critical not only for managing ARI but also for enhancing overall child health resilience across the region.

## Supplementary Information

Supplementary Material 1.

Supplementary Material 2.

## Data Availability

The data used for this study is freely available at [https://dhsprogram.com/data/available-datasets.cfm](https:/dhsprogram.com/data/available-datasets.cfm).

## Abbreviations

ARI: Acute respiratory infection
CO: Carbon monoxide
DHS: Demographic and Health Survey
HAP: Household air pollution
HSER: Household smoke exposure risk
ICF: Inner City Fund
LMICs: Low- and middle-income countries
PM: Particulate matter
SSA: sub-Saharan Africa
SSACs: sub-Saharan African countries
WHO: World Health Organization
