# Supplementary material for: Household smoke exposure risk and acute respiratory infection among children under five years in sub-Saharan Africa: evidence from the demographic and health surveys
Source: BMC Public Health. 2025 Oct 9;25:3443. doi: 10.1186/s12889-025-24708-7 (PMC12512916; doi:10.1186/s12889-025-24708-7)
Supplement: Supplementary file 1 — Supplementary Material 1. [file 12889_2025_24708_MOESM1_ESM.docx]

**Table S1. Distribution of 33 SSA countries included in the study**

| **Country** | **Frequency** | **Percent** |
| --- | --- | --- |
| Angola | 13351 | 3.46 |
| Burkina Faso | 15284 | 3.96 |
| Benin | 13449 | 3.49 |
| Burundi | 13604 | 3.53 |
| Congo Democratic Rep | 18345 | 4.76 |
| Congo Brazzaville | 8116 | 2.11 |
| Cote d’Ivoire | 7451 | 1.93 |
| Cameroun | 10059 | 2.61 |
| Ethiopia | 11007 | 2.85 |
| Gabon | 10184 | 2.64 |
| Ghana | 5684 | 1.47 |
| Gambia | 15272 | 3.96 |
| Guinea | 7880 | 2.04 |
| Kenya | 19534 | 5.07 |
| Comoros | 6427 | 1.67 |
| Liberia | 5256 | 1.36 |
| Lesotho | 3112 | 0.81 |
| Mali | 10246 | 2.66 |
| Malawi | 17394 | 4.51 |
| Mozambique | 11604 | 3.01 |
| Nigeria | 34178 | 8.87 |
| Niger | 13214 | 3.43 |
| Namibia | 4798 | 1.24 |
| Rwanda | 8324 | 2.16 |
| Sierra Leone | 19540 | 5.07 |
| Senegal | 5170 | 1.34 |
| Chad | 18535 | 4.81 |
| Togo | 13398 | 3.48 |
| Tanzania | 10043 | 2.61 |
| Uganda | 15239 | 3.95 |
| South Africa | 3570 | 0.93 |
| Zambia | 9837 | 2.55 |
| Zimbabwe | 6416 | 1.66 |
| Total | 385521 | 100.00 |

**Table S2: Pairwise correlations**

| Variables | (1) | (2) | (3) | (4) | (5) | (6) | (7) | (8) | (9) | (10) | (11) | (12) | (13) |
| --- | --- | --- | --- | --- | --- | --- | --- | --- | --- | --- | --- | --- | --- |
| (1) ARI symptoms in the 2 weeks before the survey | 1.000 |  |  |  |  |  |  |  |  |  |  |  |  |
|  |  |  |  |  |  |  |  |  |  |  |  |  |  |
| (2) Household smoke exposure risk | 0.015* | 1.000 |  |  |  |  |  |  |  |  |  |  |  |
|  | (0.000) |  |  |  |  |  |  |  |  |  |  |  |  |
| (3) Child’s sex | 0.007* | 0.000 | 1.000 |  |  |  |  |  |  |  |  |  |  |
|  | (0.000) | (0.822) |  |  |  |  |  |  |  |  |  |  |  |
| (4) Child’s age | -0.014* | 0.003* | -0.003* | 1.000 |  |  |  |  |  |  |  |  |  |
|  | (0.000) | (0.044) | (0.032) |  |  |  |  |  |  |  |  |  |  |
| (5) Child lives with mother | -0.048* | -0.023* | 0.012* | 0.097* | 1.000 |  |  |  |  |  |  |  |  |
|  | (0.000) | (0.000) | (0.000) | (0.000) |  |  |  |  |  |  |  |  |  |
| (6) Mother’s education | -0.015* | 0.122* | 0.000 | 0.015* | -0.061* | 1.000 |  |  |  |  |  |  |  |
|  | (0.000) | (0.000) | (0.952) | (0.000) | (0.000) |  |  |  |  |  |  |  |  |
| (7) Initiation of breastfeeding | 0.035* | -0.016* | 0.010* | -0.001 | 0.095* | 0.023* | 1.000 |  |  |  |  |  |  |
|  | (0.000) | (0.000) | (0.000) | (0.415) | (0.000) | (0.000) |  |  |  |  |  |  |  |
| (8) Marital status | 0.009* | 0.074* | 0.000 | 0.060* | -0.035* | 0.065* | -0.028* | 1.000 |  |  |  |  |  |
|  | (0.000) | (0.000) | (0.972) | (0.000) | (0.000) | (0.000) | (0.000) |  |  |  |  |  |  |
| (9) Mother’s age | -0.006* | 0.008* | -0.003* | 0.111* | -0.066* | 0.132* | -0.025* | 0.171* | 1.000 |  |  |  |  |
|  | (0.000) | (0.000) | (0.047) | (0.000) | (0.000) | (0.000) | (0.000) | (0.000) |  |  |  |  |  |
| (10) Wealth index | -0.024* | -0.204* | 0.004* | 0.004* | 0.012* | -0.227* | -0.021* | -0.040* | 0.020* | 1.000 |  |  |  |
|  | (0.000) | (0.000) | (0.011) | (0.013) | (0.000) | (0.000) | (0.000) | (0.000) | (0.000) |  |  |  |  |
| (11) Main floor material | -0.028* | -0.234* | 0.004* | -0.005* | 0.021* | -0.166* | 0.010* | -0.069* | 0.014* | 0.510* | 1.000 |  |  |
|  | (0.000) | (0.000) | (0.005) | (0.002) | (0.000) | (0.000) | (0.000) | (0.000) | (0.000) | (0.000) |  |  |  |
| (12) No. of children under 5 years per household | 0.002 | 0.074* | -0.006* | -0.046* | -0.248* | 0.145* | -0.030* | 0.023* | 0.074* | -0.110* | -0.095* | 1.000 |  |
|  | (0.239) | (0.000) | (0.000) | (0.000) | (0.000) | (0.000) | (0.000) | (0.000) | (0.000) | (0.000) | (0.000) |  |  |
| (13) Place of residence | 0.017* | 0.325* | -0.002 | 0.000 | -0.024* | 0.218* | -0.026* | 0.050* | 0.002 | -0.510* | -0.414* | 0.096* | 1.000 |
|  | (0.000) | (0.000) | (0.120) | (0.856) | (0.000) | (0.000) | (0.000) | (0.000) | (0.246) | (0.000) | (0.000) | (0.000) |  |
| **** p<0.01, ** p<0.05, * p<0.1* | | | | | | | | | | | | | |

**Table S3. Measurement of covariates**

| **Variables** | **Categories** |
| --- | --- |
| **Mothers’ characteristics** |  |
| Age of mother | Below 35 |
|  | 35-49 |
| Education | Below high school |
|  | High school and above |
| Marital status | Never in union |
|  | Currently in union |
|  | Formerly in union |
| **Children characteristics** |  |
| Sex of child | Male |
|  | Female |
| Current age (in months/years) | 0-12 months |
|  | 1 year and older |
| Initiation of breastfeeding | Immediately (within 1^st^ hour of after birth) |
|  | Within first day |
|  | After first day |
| Lives with mother | Yes |
|  | No |
| **Households’ characteristics** |  |
| Child ARI | No |
|  | Yes |
| Smoke exposure risk | High HSER |
|  | Medium HSER |
|  | Low HSER |
|  | Very Low HSER |
| Wealth status | Poor |
|  | Middle |
|  | Rich |
| Place of residence | Urban |
|  | Rural |
| Number of children under 5 years per household | One child |
|  | More than one child |
| Main floor material | Unimproved |
|  | Improved |

**Table S4: Descriptive Statistics**

| Variable | N | Mean | Std. Dev. | Min | Max |
| --- | --- | --- | --- | --- | --- |
| ARI symptoms in the 2 weeks before the survey | 390987 | .042 | .2 | 0 | 1 |
| Household smoke exposure risk | 391438 | 1.526 | .663 | 1 | 4 |
| Child’s sex | 403734 | 1.494 | .5 | 1 | 2 |
| Child’s age | 377304 | .786 | .41 | 0 | 1 |
| Child lives with mother | 417417 | .137 | .344 | 0 | 1 |
| Mother’s education | 417395 | .395 | .489 | 0 | 1 |
| Initiation of breastfeeding | 417417 | 1.68 | .758 | 1 | 3 |
| Mother’s Age | 417417 | 1.947 | .725 | 1 | 3 |
| Marital status | 417417 | .996 | .376 | 0 | 2 |
| Wealth index | 417417 | 1.85 | .884 | 1 | 3 |
| Main floor material | 417352 | 1.459 | .498 | 1 | 2 |
| No. of children under 5 yrs per household | 417417 | .649 | .477 | 0 | 1 |
| Place of residence | 417417 | 1.686 | .464 | 1 | 2 |
