# Supplementary material for: Household smoke exposure risk and acute respiratory infection among children under five years in sub-Saharan Africa: evidence from the demographic and health surveys
Source: BMC Public Health. 2025 Oct 9;25:3443. doi: 10.1186/s12889-025-24708-7 (PMC12512916; doi:10.1186/s12889-025-24708-7)
Supplement: Supplementary file 2 — Supplementary Material 2. [file 12889_2025_24708_MOESM2_ESM.docx]

**Table S5. Effect of HSER on Child ARI infections (marginal effects)**

|  | (1) | (2) | (3) | (4) |
| --- | --- | --- | --- | --- |
| Variables | marginal effects | aster | ci | se |
| Smoke Exposure Risk==Low (RC) |  |  |  |  |
| Smoke Exposure Risk==Medium | 0.001 |  | -0.002 - 0.004 | 0.001 |
| Smoke Exposure Risk==High | 0.009 | *** | 0.006 - 0.011 | 0.001 |
|  |  |  |  |  |
| Observations | 365,901 |  |  |  |
| Dependent Variable | Child ARI Infections |  |  |  |
| Wald chi2 | 509*** |  |  |  |
| Pseudo R2 | 0.004 |  |  |  |
| Regional Fixed Effects | Yes |  |  |  |

RC = Reference category; ci = 95% confidence intervale; se= Standard errors.

*** p<0.01, ** p<0.05, * p<0.1

**Table S6. Effect of HSER, child, mother and household characteristics on Child ARI infections (marginal effects)**

|  | (1) | | (2) | | (3) | | (4) | |  |
| --- | --- | --- | --- | --- | --- | --- | --- | --- | --- |
| Variables | marginal effects | | aster | | ci | | se | |  |
| Smoke Exposure Risk==Low (RC) |  | |  | |  | |  | |  |
| Smoke Exposure Risk==Medium | -0.002 | | * | | -0.005 - 0.000 | | 0.001 | |  |
| Smoke Exposure Risk==High | 0.003 | | ** | | 0.001 - 0.006 | | 0.001 | |  |
| Sex of Child==Male (RC) | |  | |  | |  | |  | |
| Sex of Child==Female | | 0.003 | | *** | | 0.001 - 0.004 | | 0.001 | |
| Child’s age==0 (0-12 months) (RC) | |  | |  | |  | |  | |
| Child’s age==1 (1 year or more) | | -0.006 | | *** | | -0.007 - -0.004 | | 0.001 | |
| Lives with mother== Yes (1) | | -0.046 | | *** | | -0.052 - -0.041 | | 0.003 | |
| Lives with mother== No (0) (RC) | |  | |  | |  | |  | |
| Education of mother ==0 (Below high school) (RC) | |  | |  | |  | |  | |
| Education of mother ==1 (High school and above) | | -0.012 | | *** | | -0.014 - -0.011 | | 0.001 | |
| Initiation of breast==1 (Immediately) (RC) | |  | |  | |  | |  | |
| Initiation of breast==2 (within first day) | | 0.011 | | *** | | 0.009 - 0.012 | | 0.001 | |
| Initiation of breast==3 (after first day) | | 0.020 | | *** | | 0.018 - 0.022 | | 0.001 | |
| Marital status==0 (Never in union) (RC) |  | |  | |  | |  | |  |
| Marital status==1 (union/living with a man) | 0.004 | | *** | | 0.002 - 0.007 | | 0.001 | |  |
| Marital status==2 (formerly in union) | 0.008 | | *** | | 0.005 - 0.012 | | 0.002 | |  |
| Age of mother==1 (15 – 24 years) (RC) |  | |  | |  | |  | |  |
| Age of mother==3 (35 – 49 years) | -0.002 | | *** | | -0.004 - -0.001 | | 0.001 | |  |
| Wealth index ==1 (Poor) (RC) |  | |  | |  | |  | |  |
| Wealth index ==2 (Middle) | -0.003 | | *** | | -0.005 - -0.001 | | 0.001 | |  |
| Wealth index ==3 (Rich) | -0.005 | | *** | | -0.007 - -0.003 | | 0.001 | |  |
| Main floor material ==1 (Unimproved) (RC) |  | |  | |  | |  | |  |
| Main floor material ==2 (improved) | -0.010 | | *** | | -0.012 - -0.008 | | 0.001 | |  |
| No. of children under 5 in household == 1 (One child) (RC) |  | |  | |  | |  | |  |
| No. of children under 5 in household == 2 (More than one) | -0.004 | | *** | | -0.006 - -0.003 | | 0.001 | |  |
| Place of residence ==1 (Urban) (RC) |  | |  | |  | |  | |  |
| Place of residence ==2 (rural) | 0.003 | | *** | | 0.001 - 0.005 | | 0.001 | |  |
| Observations | 365,830 | |  | |  | |  | |  |
| Dependent Variable | Child ARI Infections | |  | |  | |  | |  |
| Wald chi2 | 2025*** | |  | |  | |  | |  |
| Pseudo R2 | 0.0165 | |  | |  | |  | |  |
| Regional Fixed Effects | Yes | |  | |  | |  | |  |

RC = Reference category; ci = 95% confidence intervale; se= Standard errors.

*** p<0.01, ** p<0.05, * p<0.1
